# Conducting simulation studies for computerized adaptive testing using SimulCAT: an instructional piece

**DOI:** 10.3352/jeehp.2018.15.20

**Published:** 2018-08-17

**Authors:** Kyung (Chris) Tyek Han

**Affiliations:** Graduate Management Admission Council, Reston, VA, USA; Hallym University, Korea

**Keywords:** Adaptive Testing, Simulation, Computer software

## Abstract

Computerized adaptive testing (CAT) technology is widely used in a variety of licensing and certification examinations administered to health professionals in the United States. Many more countries worldwide are expected to adopt CAT for their national licensing examinations for health professionals due to its reduced test time and more accurate estimation of a test-taker’s performance ability. Continuous improvements to CAT algorithms promote the stability and reliability of the results of such examinations. For this reason, conducting simulation studies is a critically important component of evaluating the design of CAT programs and their implementation. This report introduces the principles of SimulCAT, a software program developed for conducting CAT simulation studies. The key evaluation criteria for CAT simulation studies are explained and some guidelines are offered for practitioners and test developers. A step-by-step tutorial example of a SimulCAT run is also presented. The SimulCAT program supports most of the methods used for the 3 key components of item selection in CAT: the item selection criterion, item exposure control, and content balancing. Methods for determining the test length (fixed or variable) and score estimation algorithms are also covered. The simulation studies presented include output files for the response string, item use, standard error of estimation, Newton-Raphson iteration information, theta estimation, the full response matrix, and the true standard error of estimation. In CAT simulations, one condition cannot be generalized to another; therefore, it is recommended that practitioners perform CAT simulation studies in each stage of CAT development.

## Introduction

As of 2018, 4 institutions in the United States have widely incorporated computerized adaptive testing (CAT) technology in highstakes examinations for U.S. health professionals: (1) the National Council of State Boards of Nursing for the National Council Licensure Examination for Registered Nurses; (2) the American Society for Clinical Pathology Board of Certification for 24 examinations, including the Medical Laboratory Scientist, Medical Laboratory Technician, Phlebotomy Technician, and Histotechnician examinations; (3) the National Registry of Emergency Medical Technicians for the Emergency Medical Responder, Emergency Medical Technician (EMT), and Advanced-EMT exams; and (4) the National Association of Boards of Pharmacy for the North American Pharmacist Licensure Examination. The stability of the CAT-based exams administered by these institutions has been maintained due to continual improvements to the CAT algorithm. It is anticipated that many more countries worldwide will adopt CAT administration for national licensing examinations for health professions due to the reduced test time and more accurate estimation of the test-taker’s ability parameter. Furthermore, re-testing can be accomplished more easily with CAT than with other forms of computer-based testing or paper-and-pencil testing.

When taking a CAT-based exam, a test-taker’s performance is evaluated not just at the end of the test, but continuously during the testing period—specifically, after the administration of each item. Based on the interim evaluation of the test-taker’s performance, the CAT engine selects and administers the next test item at the difficulty level expected to be the most relevant to the test-taker’s performance ability. Such an individualized adaptive test construct can dramatically increase the efficiency of the measurement tool [1]. It is not unusual to see test length decrease by 30% to 50% with CAT administration, while maintaining a similar level of test score accuracy.

Because test forms are assembled on-the-fly in CAT, the CAT development process is completely different from that used for constructing linear, fixed test forms. In typical fixed test-form construction, the created test form is usually evaluated analytically based on, for example, a test score reliability index, the standard error of measurement, the test information function (if operating in the framework of item response theory), and many other criteria. With CAT, the number of possible adaptive forms grows exponentially as the size of the item pool and test length increase. For example, if one administers CAT with 30 items using an item pool of 500 items, the number of possible test forms would be approximately 3.83359E+80. This would make the evaluation of CAT test forms using standard analytical means impractical, if not impossible.

Simulation techniques using the Monte-Carlo (MC) method have been used extensively in the field of educational measurement. The known true values of MC simulations, such as the person parameter (*θ*) and item parameters, make studies of test construction, item analysis, dat*a*-model fit evaluation, differential item function, test performance and score distribution, and others not only possible but effective. The MC simulation method is especially important in the CAT arena because often it is the only practical way to study and evaluate CAT programs and their implementation. As noted above, other analytical methods are often not applicable or feasible with CAT programs.

SimulCAT is a software program that was developed as a solution for CAT simulation in response to strong demand for an easy-touse, comprehensive tool for performing such simulations [2]. The program is freely available from: https://www.umass.edu/remp/software/simcata/simulcat/. This article introduces the basics of Simul-CAT, as well as some key statistics for evaluating simulation results, followed by several examples of simulation studies.

## Basics of SimulCAT

SimulCAT implements all 3 (iterative) processes of CAT as explained by Han [1]: (1) *θ* estimation, (2) CAT termination policy, and (3) item selection. It supports various CAT administration options to create CAT testing environments that are as realistic as possible. The interim and final score estimates can be c1a6lc ulated using the maximum likelihood, the Bayesian maximum a posteriori or expected a posteriori estimations, or the maximum likelihood estimation with fences [3]. SimulCAT users also can set the initial score value, the range of score estimates, and restrictions on how much the estimate can change. The length of CAT administration can be either fixed or variable. For variable-length testing, SimulCAT supports multiple termination rules, including the standard error of estimation (SEE) and score estimate consistency. Within SimulCAT, users can choose the number of test takers to be administered simultaneously at each test time slot and set the frequency of communication between a test server and client computers (i.e., terminals).

The core part of CAT is the item selection process, and Simul-CAT supports most of the methods described by Han [1] for the 3 key components of item selection: the item selection criterion, item exposure control, and content balancing. The item selection criteria supported in SimulCAT include (but are not limited to): (1) maximized Fisher information (MFI) [4], (2) *a*-stratification [5,6], (3) global information [7], (4) interval information [8], (5) likelihood weighted information [8], (6) gradual maximum information ratio [9], and (7) efficiency balanced information [10].

Along with a choice of item selection criteria, a variety of item exposure control options are available within SimulCAT, including the following: (1) randomesque strategy [11], (2) Sympson and Hetter [12] method, (3) multinomial methods, both conditional and unconditional [13,14], and (4) fade-away method [9].

For content balancing, SimulCAT supports the content script method and the constrained CAT method [11].

**Fig. 1.**
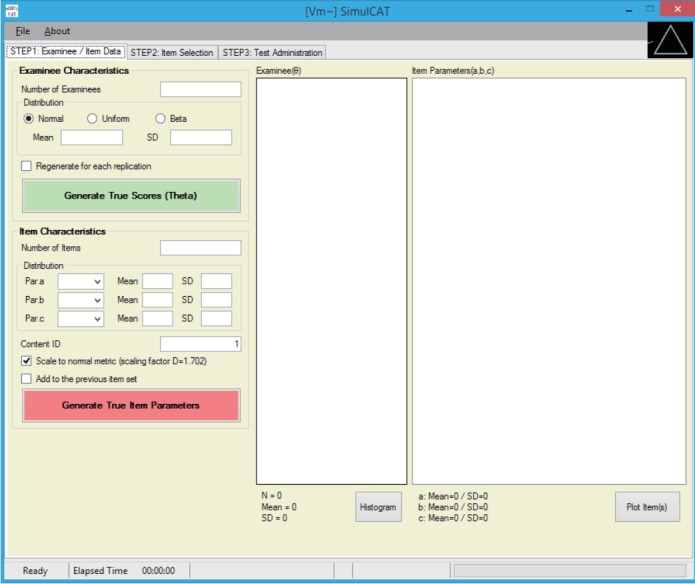
SimulCAT GUI step 1: examinee/item data. Users can generate examinee and item data from normal, uniform, and beta distributions. SimulCAT also supports charts of the generated examinee and item data (the “Histogram” button and “Plot Item(s)” button). GUI, graphical user interface.

**Fig. 2.**
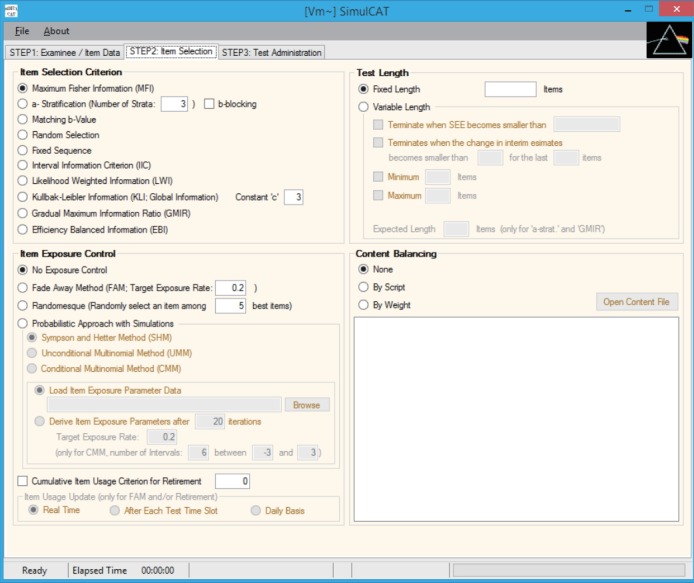
SimulCAT GUI step 2: item selection. The software prompts users to choose item selection criteria, item exposure control, test length, and content balancing. GUI, graphical user interface.

**Fig. 3.**
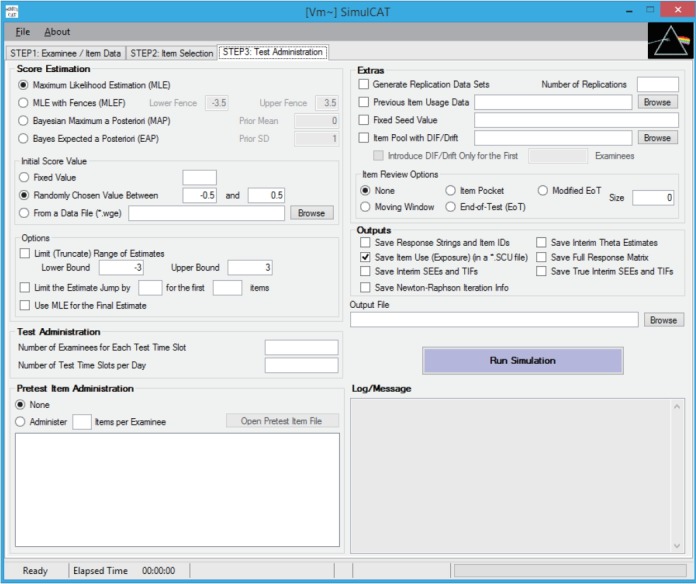
SimulCAT GUI step 3: test administration. Users specify the details of the testing environment of computerized adaptive testing, for example, the score estimation method, number of simultaneous test administrations, pretest item administration, and other research tools and output options. GUI, graphical user interface.

SimulCAT features an intuitive graphical user interface (GUI) that involves a 3-step process: (1) examinee and item data generation, (2) item selection, and (3) test administration. Figs. 1–3 display the software prompts for each of the 3 steps.

Software users may choose to use a syntax file mode and/or batch mode instead of SimulCAT’s GUI, and also may use existing examinee and item data sets.

**Fig. 4.**
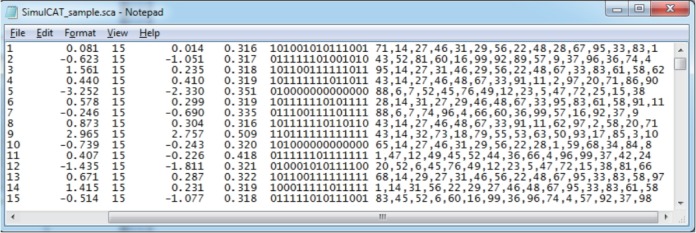
SimulCAT output file 1: SimulCAT test administration data (*.sca). Each column shows the examinee’s ID, true theta value, test length, final theta estimate, the standard error of estimation, responses, and item ID, respectively.

**Fig. 5.**
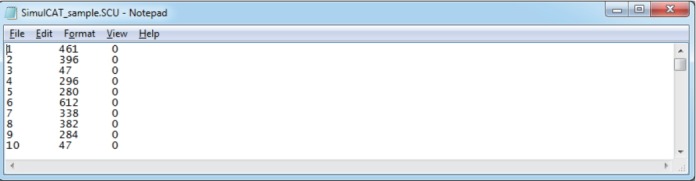
SimulCAT output file 2: SimulCAT item use data (*.scu). Each column shows the item ID, item exposure, and number of days after item retirement, respectively.

Once SimulCAT completes a simulation run (or a set of replications), it saves the following 2 output files by default (Figs. 4, 5).

SimulCAT software, user’s manual, and sample files can be downloaded from the website at http://www.hantest.net. MSTGen [15] is also available for simulations involving multistage testing [16].

## Some statistics for evaluating computerized adaptive testing performance

Once a test program based on CAT is designed, it is important to evaluate the CAT administration’s performance, given the item pool and test-taker distribution, using a CAT simulation. The most important aspect of CAT performance evaluation is the measurement precision of the test program. Except for CAT applications such as patient self-report surveys and personality assessments, where individuals’ pre-knowledge of test items would not change their response behavior, it is often equally important to evaluate the performance of most CAT programs regarding test security, especially for tests with high-stakes consequences, as in college admissions. The next section introduces several useful statistics and indices for evaluating CAT performance.

### Measures of measurement precision

Because true *θ* values are readily available in MC simulation studies, evaluating the measurement error and precision of CAT is a straightforward calculation. The bias statistic, which is a measure of systematic measurement errors, can be computed simply by averaging the difference between the estimated *θ* ($\hat{\theta }$) and true *θ* across all test takers (a.k.a., simulees). That is

(1) $$\mathrm{Bias}\;=\;\frac{\sum_{i=1}^{I}\left({\hat{\theta }}_{i}-\theta_{i}\right)}{I}$$

where *I* is the number of simulees. The bias statistic is commonly used with most conventional, nonadaptive test programs, and is still often used with many CAT programs as a “good-to-know” summary statistic. However, because the characteristic of test forms can differ dramatically across *θ* ranges with CAT, conditional bias (CBIAS), which is bias within each *θ* range (for example, *θ*< −2, −2≤ *θ*< −1, −1≤ *θ*< 0, 0≤ *θ*< 1, 1≤ *θ*< 2, and *θ*≥ 2), is generally the more important statistic to investigate in CAT. An example of CBIAS will be presented later in this article.

The mean absolute error (MAE) is a useful statistic that summarizes overall measurement error (both systematic and nonsystematic error). The MAE can be computed as

(2) $$\mathrm{MAE}\;=\;\frac{\sum_{i=1}^{I}\left|{\hat{\theta }}_{i}-\theta_{i}\right|}{I}$$

The root mean square error (RMSE) is another frequently used statistic, and is expressed as

(3) $$\mathrm{RMSE}\;=\;\sqrt{\frac{\sum_{i=1}^{I}{\left({\hat{\theta }}_{i}-\theta_{i}\right)}^{2}}{I}}$$

As in the example of bias, when using the MAE and RMSE statistics with a CAT simulation, it is often more meaningful to examine the conditional MAE (CMAE) and conditional RMSE (CRMSE) for each *θ* range.

The CMAE and CRMSE across different *θ* ranges are often interpreted as approximates of the conditional standard error of measurement (CSEM). The exact point estimate of CSEM can be easily computed using the CRMSE with a simulation design that repeats thousands of simulees at the same exact *θ* value; for example, for 1,000 simulees with *θ*= −1, the CRMSE of those 1,000 simulees is the CSEM at *θ*= −1.

The SEE at the final $\hat{\theta }$, which is $1/\sqrt{TIF}$, where TIF stands for the test information function for the specific test form that each simulee was administered, is often a readily available statistic in CAT. It is sometimes used as a CAT termination criterion for variable-length CAT programs. Like CMAE and CRMSE, the conditional SEE (CSEE) also is often interpreted as an approximation of the CSEM; however, it should be noted that the relationship between the CSEE and the actual CSEM may not be consistent, depending on other factors such as test length and the *θ* estimation method.

The reliability coefficient was one of most extensively used indices for evaluating the quality of test programs in the classical test theory era. It can sometimes still be appropriate to use the reliability coefficient for CAT-based programs; however, as pointed out above, the characteristics of CAT test forms can vary dramatically across *θ* ranges, and the reliability coefficient could often mislead people about the quality of measurement at different *θ* levels. For CAT programs, reporting the CSEM (or its approximations, such as the CRMSE or CMAE) at the most relevant *θ* ranges is strongly advised instead of reporting the reliability coefficient. If the reliability coefficient must be reported, it should be computed based on the most representative sample set of simulees, and it should be accompanied by CSEM statistics.

If a CAT program is of variable length, the CSEE is usually tightly controlled as a CAT termination criterion, and it is important to evaluate the conditional test length as an indicator of measurement efficiency.

### Measures for test security performance

Most measures used to evaluate test security performance in CAT focus on item exposure because in CAT items are reused over time, and highly exposed items are likely to increase the chance of test takers obtaining knowledge about those items. Compromised test security could pose serious threats to test validity and fairness, since test takers who gain pre-knowledge about the compromised items would respond to the items differently. When simulation studies are conducted to evaluate the test security aspects of CAT, it is extremely important to use a sample set of simulees that reflects the actual testtaker population.

The maximum item exposure rate is directly observable from the item usage file (*.scu) of SimulCAT. The maximum item exposure rate often forms the basis of exposure control methods such as the Sympson and Hetter [12] method. Even if the overall maximum item exposure rate is tightly controlled at a certain target, it is often possible to have a much higher item exposure rate to test takers at a similar *θ* level. Therefore, it is common for high-stakes tests to evaluate and control the conditional maximum item exposure within each *θ* level [14].

The percentage of unused test items offers important insights into the efficiency of the CAT algorithm and is sometimes (especially with a small item pool) indirectly related to overexposure of some items. A large percentage of unused items often indicates that the item pool was not optimally designed for the specific CAT algorithm given the size of the test-taker population.

The standard deviation of the item exposure rate is another useful statistic for understanding the overall effectiveness of item exposure control and pool utilization. For example, if 2 different CAT designs result in similar CSEMs, then the CAT design that exhibits a lower standard deviation of the item exposure rate (i.e., a more even use of items) should be selected.

The average percentage of test overlap among all pairs of observed test forms is another test security-related measure that is often reported for CAT-based programs. It can be computed by

(4) T = p∑j=1Jrj2kp-1-1p-1'

where J is the number of items in the pool, *r_j_* is the exposure of item *j, p* is the number of (fixed-length) CAT forms administered, and k is the number of items in each form. Because this test overlap index is used frequently in practice, it could cause test practitioners to overlook the worst instances of test overlap. Thus, test practitioners should still investigate the most severe cases of test overlap, even if the average between-test overlap index is within a reasonable range.

Computing the correlation coefficient between items’ *a*-parameter values and item exposure could provide important information about the pattern of item selection and use given the item selection criterion of CAT. A test item’s *a*-parameter is one of the most important factors in the item information function, and many item selection criteria, including the MFI, have a strong tendency toward excessive use of items with higher *a*-parameter values. If the correlation coefficient is observed to be too high to accept, a test practitioner might improve the situation by lowering the target item exposure rate or changing the item selection criterion (for example, from the MFI to the *b*-matching method).

## Example of a computerized adaptive testing simulation study using SimulCAT

**Fig. 6.**
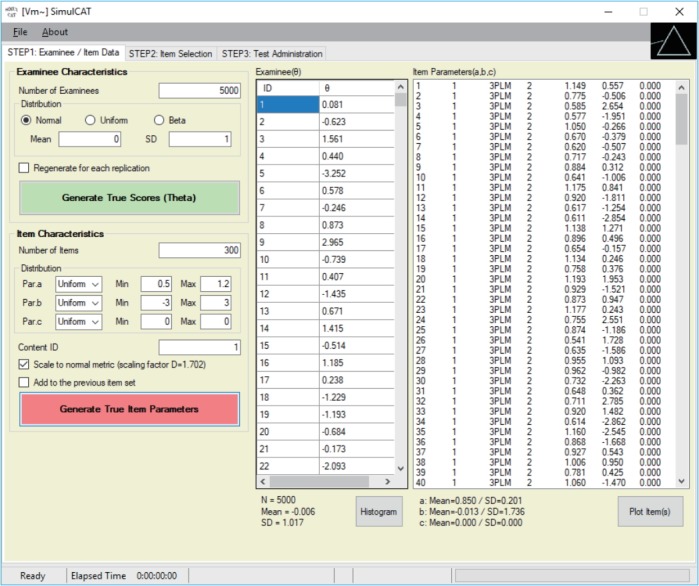
Display of 5,000 simulees randomly drawn from a standard normal distribution (actual θ values differ each time), and 300 items generated for the item pool (item parameters differ each time, and are generated by clicking on the “Generate True Item Parameters” button).

**Fig. 7.**
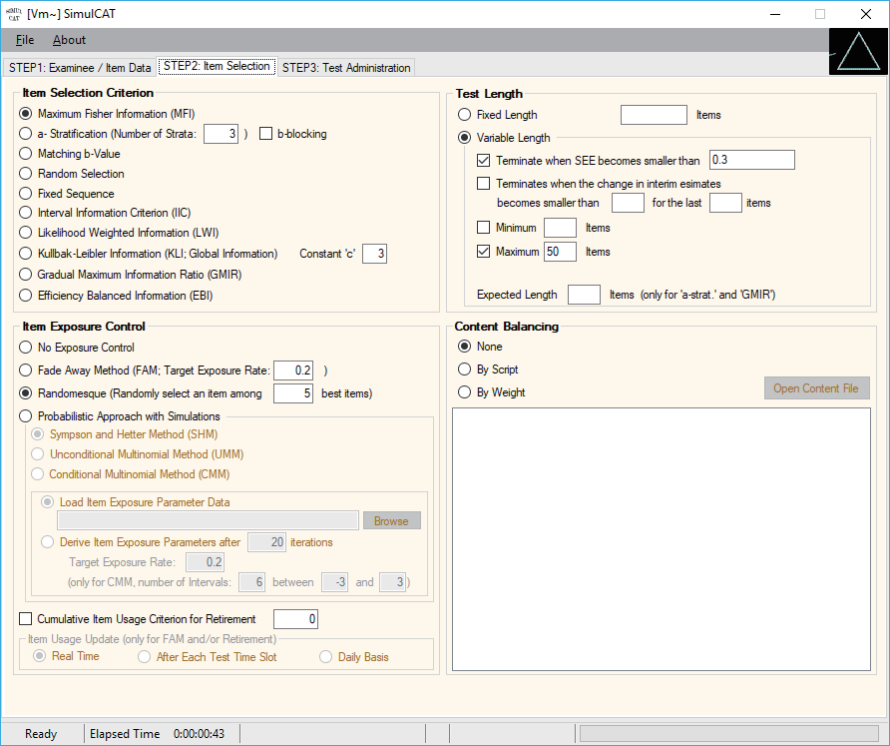
Input for step 2.

**Fig. 8.**
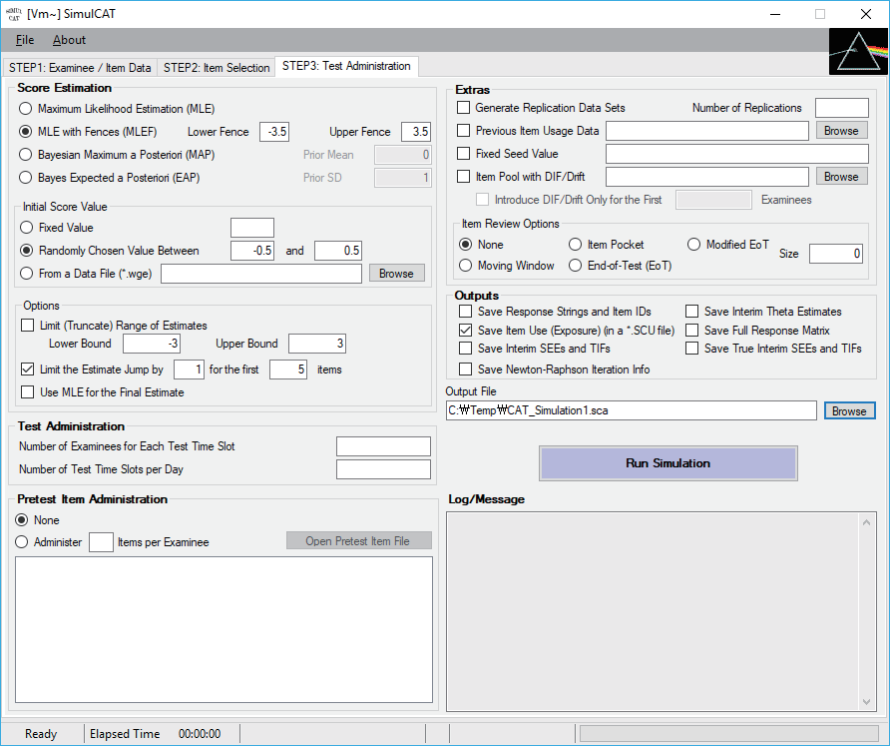
Input for step 3. After filling in all necessary information, including the output file name, the user clicks on the “Run Simulation” button.

The CAT design of this example is presented in Table 1. The input values that should be recorded in the SimulCAT GUI are presented in Figs. 6–8.

**Fig. 9.**
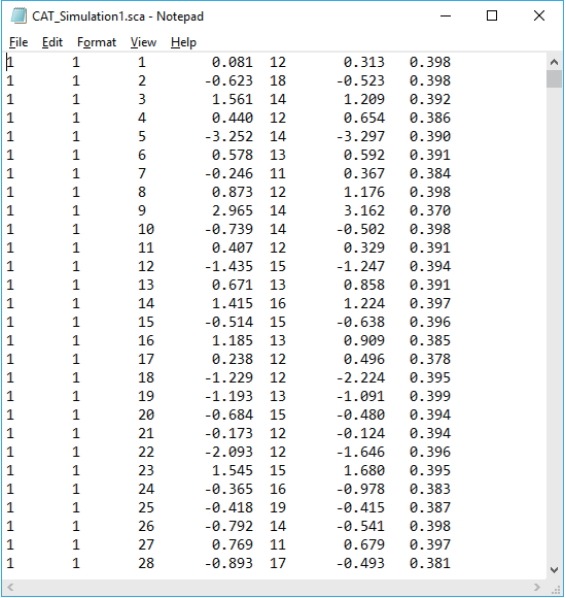
SimulCAT output file with test administration information (*.sca). The values in columns 3 through 7 indicate the simulee ID, true θ, number of items administered, θ estimate, and standard error of estimation, respectively.

**Fig. 10.**
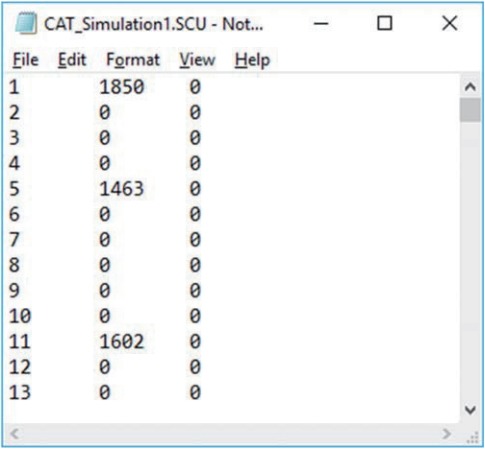
SimulCAT output file with information about item exposure/usage. The first 2 columns indicate the item ID and number of item exposures, respectively.

**Fig. 11.**
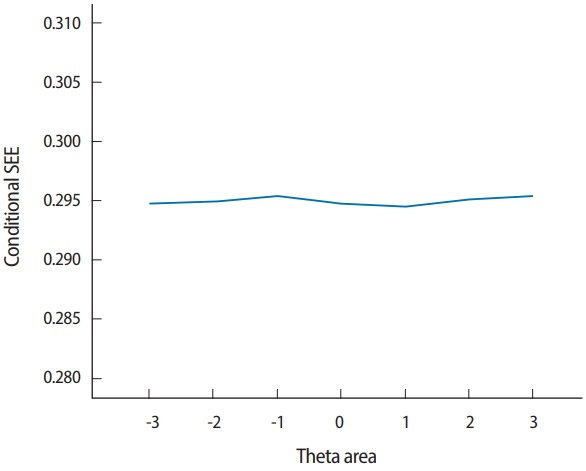
Conditional SEE (average SEE in each θ range). SEE, standard error of estimation.

**Fig. 12.**
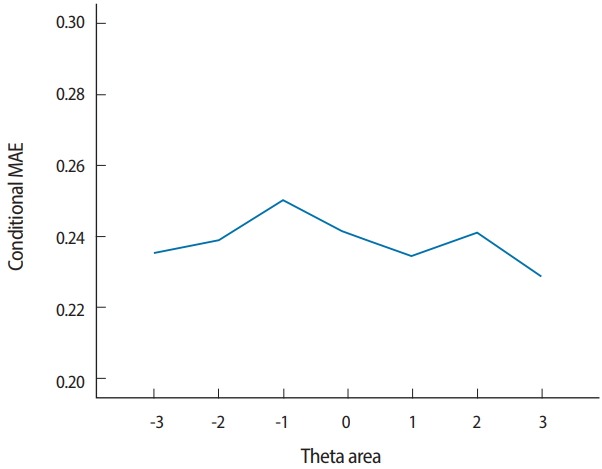
Conditional MAE (average MAE in each θ range). MAE, mean absolute error.

**Fig. 13.**
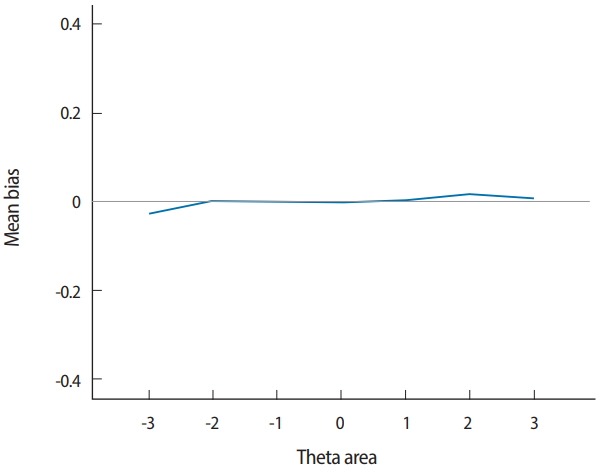
Conditional bias (average bias within each θ range).

**Fig. 14.**
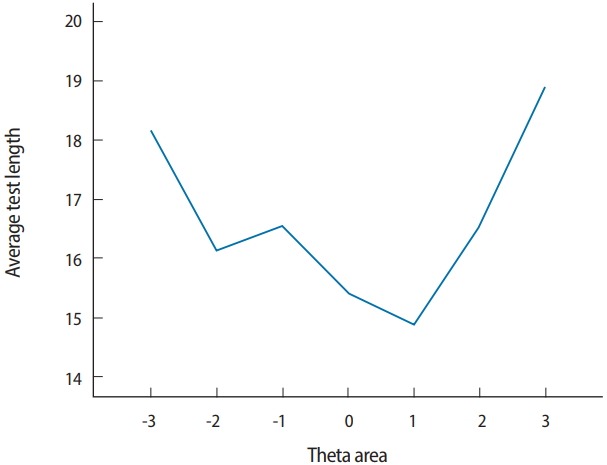
Average test length in each θ range.

Two output files are generated after running SimulCAT, as shown in Figs. 9 and 10. It is important to note that the actual values shown in Figs. 9 and 10 differ with each operation because of the random nature of MC simulation. Nonetheless, the findings of the simulation should be consistent across replications. Using the values (true *θ*, *θ* estimate, and SEE) in the SimulCAT output file (*.sca), we can easily compute and plot CSEE, CMAE, and CBIAS (SPSS was used in this example). The simulation results show that the CSEE was tightly controlled to be lower than 0.3 across all *θ* areas as targeted (Fig. 11), and the actual observed errors based on CMAE seemed to be consistent across all *θ* areas (Fig. 12). The CBIAS (Fig. 13) indicated that the observed systematic error was almost zero. The average number of items administered (while satisfying the SEE criterion of 0.3) was less than 17 when −2<*θ*<2 (Fig. 14). The average test length increased to about 18 when $\left|\theta \right|$ > 3, which was within the expected range for this CAT design where the initial *θ* value was set to be chosen randomly between −1 and 1, and the *θ* estimate jump was limited to less than 1 for the first 5 items. If one wants to reduce the average number of items for individuals at the extreme *θ* level, one could consider relaxing the constraint for the *θ* estimate jump.

**Fig. 15.**
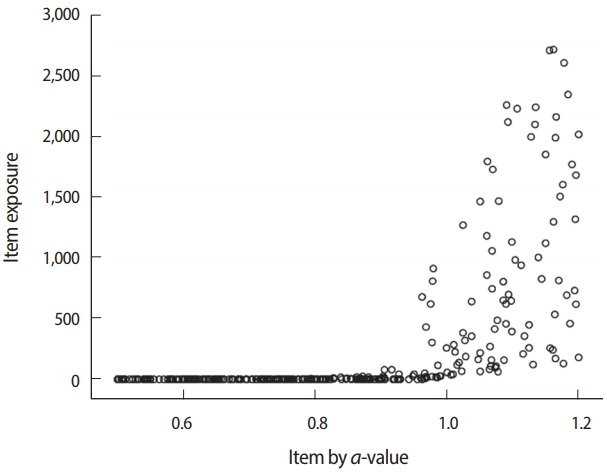
Item exposure by *a*-value.

**Fig. 16.**
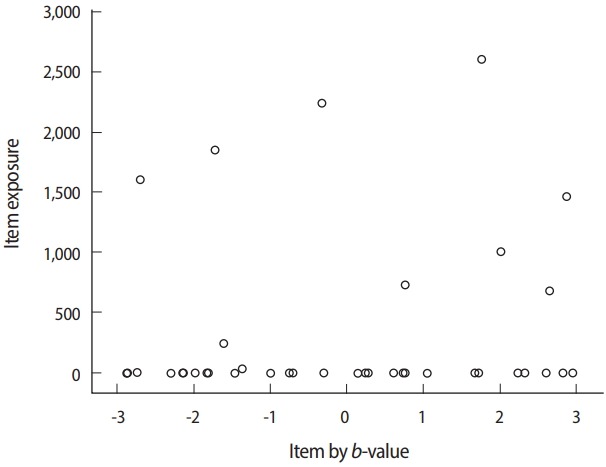
Item exposure by *b*-value.

The evaluation of item exposure control and item pool is mainly accomplished by using the item usage output file (*.scu) of Simul-CAT. The maximum observed item exposure rate exceeded 2,700 (out of 5,000 test administrations/simulees), meaning that the randomesque method and its setting (1 of the best 5 items) was not sufficiently effective. Moreover, 164 of the 300 items (54.7% of the item pool) were not used at all. Looking deeper into the situation by investigating the relationship between item exposure and *a*-parameter values (Fig. 15), it is apparent that the studied CAT design too strongly favored items with higher *a*-values. One possible change to remedy this issue would be changing the item selection criterion from the MFI method to the *b*-matching method, which does not take *a*-values into consideration. Regarding the item difficulty (*b*-value) of items in the pool, there seemed to be no shortage of items with certain difficulty levels (Fig. 16).

This purpose of this CAT simulation study demonstrates how a representative CAT simulation study can be conducted and how its results can be interpreted. In many cases, the simulation process and analyses of results shown in this example can be a good starting point for a CAT simulation study. This example, nevertheless, should not be understood as a rule of thumb for CAT simulation study practice, since each CAT simulation study examines different aspects of CAT depending on the research question.

## Conclusion

Conducting simulation studies is essential in almost all stages of CAT development, namely: the test blueprint and target planning stage, the CAT algorithm development stage, the item pool construction stage, and the quality control/assurance stage. To make simulation studies as effective as possible, it is critically important to clearly identify the research questions and to design the CAT simulation accordingly. It should also be noted that in many CAT simulations, the findings are often not generalizable to other conditions. For example, even when the same CAT design and specifications are repeated, a couple of item changes in the item pool could completely alter the item exposure rates (especially if the Sympson and Hetter method is used). For this reason, CAT simulations should be repeated in each stage of CAT development.

## Figures and Tables

**Table 1.** Example of computerized adaptive testing simulation design

Simulees	10,000 Simulees with θ–N (0, 1)
Item pool	300 Items based on a 2-parameter logistic item response theory model
	• ma–U (0.5, 1.2)
	• b–U (-3, 3)
Item selection criterion	Maximum Fisher information
Item exposure control	Randomesque (randomly select an item from among the 5 best items)
Test length	Variable length
	• aTerminate when standard error of estimation becomes smaller than 0.3
	• bMaximum of 50 items
Content balancing	None
Score estimation	maximum likelihood estimation with fences (lower and upper fences at −3.5 and 3.5, respectively)
	Initial score randomly chosen between −0.5 and 0.5
	Limit the estimate jump by 1 for the first 5 items
Outputs	Save item use (exposure)

## Supplementary material

Supplement 1. Audio recording of the abstract.
